# Development of a predictive model based on ultrasonographic features of brucella-associated periarthritis of the shoulder

**DOI:** 10.3389/fcimb.2026.1808903

**Published:** 2026-06-29

**Authors:** Guohua Wu, Ping Li, Lingxu Cui, Xi Qi, Fangfang Guo, Fujia Kang, Shuyu Zhang, Ni Wei

**Affiliations:** 1 Ultrasound Medicine Center, The Second Affiliated Hospital of Inner Mongolia Medical University, Hohhot, China; 2 Graduate School, Inner Mongolia Medical University, Hohhot, China

**Keywords:** arthritis, brucellosis, infection, shoulder, ultrasound

## Abstract

**Purpose:**

To investigate ultrasonographic characteristics of shoulder involvement in brucellosis and explore correlations between imaging features and shoulder pain for predictive model development.

**Materials and methods:**

A total of 111 patients with confirmed brucellosis were enrolled and divided into a shoulder pain group (n=60) and a control group without shoulder symptoms (n=51). All underwent standardized shoulder ultrasound performed by experienced operators blinded to clinical data. Ultrasonographic findings were compared between groups. Correlation analysis and Partial Least Squares (PLS) regression identified key predictors. Predictive models were evaluated using ROC analysis, nomogram visualization, and decision curve analysis (DCA).

**Results:**

Compared with the non-shoulder pain group, patients with shoulder pain showed significantly higher frequencies of subacromial-subdeltoid (SASD) bursal effusion (58.3% vs. 5.9%), long head of the biceps tendon (LHBT) sheath effusion (68.3% vs. 15.7%), and rotator cuff hypervascularity (38.3% vs. 11.8%) (all P<0.05). PLS regression identified SASD bursal thickness (≥2.2 mm), SASD effusion, and LHBT sheath effusion as key predictors. The three-variable model demonstrated the highest sensitivity (0.967), while the five-variable model achieved the best overall performance with an area under the curve of 0.957 and specificity of 0.902. Nomogram and decision curve analysis further demonstrated the model’s potential clinical utility in identifying shoulder involvement in brucellosis.

**Conclusion:**

Key ultrasonographic features are strongly associated with shoulder pain in brucellosis and provide high diagnostic value for early identification of shoulder involvement.

## Introduction

Brucellosis is a globally prevalent zoonotic disease primarily transmitted through contact with infected animals or ingestion of contaminated animal products such as unpasteurized dairy or undercooked meat. Despite being under control in many developed countries, brucellosis remains endemic in numerous regions, particularly among populations engaged in livestock farming, posing a significant public health burden (Laine et al., 2023). One of the most common complications of brucellosis is musculoskeletal involvement, of which spinal involvement is the most frequent form (Guenifi et al., 2025). While spinal involvement is most commonly reported, peripheral joints such as the shoulder may also be affected but are often under-recognized. Among these, shoulder periarthritis is an important clinical manifestation that may significantly affect patients’ quality of life. After infection, Brucella organisms can invade the shoulder joint and surrounding soft tissues, leading to local inflammation, pain, swelling, and restricted range of motion. In advanced stages, functional impairment of the shoulder joint may occur (Yu et al., 2022; Zangeneh et al., 2023). The standard treatment is early administration of combination antimicrobial therapy at sufficient dosage and duration, which can prevent progression to chronic disease or recurrence (Unuvar et al., 2019). However, except for brucellar spondylitis, there is currently a lack of standardized treatment protocols for peripheral joint involvement. The main challenges include difficulty in early identification, absence of pathological confirmation, and lack of evidence-based therapeutic guidance. Musculoskeletal ultrasound (MSK US), as a real-time, non-invasive, and cost-effective imaging modality, has been widely applied in the diagnosis and monitoring of shoulder joint disorders. In brucellosis-related shoulder periarthritis, ultrasound can detect early signs of inflammation and joint structural damage, potentially improving diagnostic accuracy and guiding treatment (Schamberger et al., 2024). This study aims to compare the ultrasonographic features of brucellosis patients with shoulder pain and those without. Through identification of key imaging differences and their correlation with shoulder pain, we intend to develop a predictive diagnostic model based on ultrasound characteristics. The ultimate goal is to facilitate early diagnosis, guide individualized treatment strategies, and improve patient outcomes.

## 1 Materials and methods

This was a prospective cross-sectional study with both descriptive analysis and predictive modeling components.

### 1.1 Clinical data collection

From August 2023 to May 2024, patients diagnosed with brucellosis at two specialized infectious disease institutions in northern China were recruited consecutively. All patients were diagnosed according to the Chinese Diagnostic Criteria for Brucellosis (WS 269-2019), based on positive serological tests and compatible clinical symptoms.

1. Inclusion criteria:
  - Aged 25–55 years;
  - Previously healthy with no history of shoulder pain before disease onset;
  - Completed clinical records;
  - Able to cooperate with shoulder MSK-US;
  - Provided written informed consent.
2. Exclusion criteria:
  - Incomplete clinical or imaging data;
  - Congenital or acquired shoulder joint deformities;
  - History of shoulder periarthritis or rotator cuff injuries before brucellosis;
  - Other systemic inflammatory diseases (e.g., gout, rheumatoid arthritis);
  - Long-standing unexplained shoulder pain prior to diagnosis;
  - Poor image quality or inability to complete ultrasound.

#### 1.1.1 Shoulder pain evaluation and grouping

Shoulder involvement was determined based on confirmed systemic brucellosis (Altunçekiç Yildirim et al., 2022), local symptoms, and corresponding ultrasonographic findings, as reported in previous studies (Elzein and Sherbeeni, 2016; Çatal, 2019). Shoulder pain was assessed using the Wong-Baker Faces Pain Scale–Revised (FPS-R, 2001 edition), which quantifies pain on a 0–10 scale (Pathak et al., 2018). This scale has been validated in middle-aged and older populations with lower educational attainment (Lor et al., 2021), characteristics that match our patient cohort. Brucellosis patients reporting recent shoulder discomfort were divided into two groups based on their FPS-R scores. Those with scores ≥4 were assigned to the Shoulder Pain Group (SPG), indicating at least mild pain in the shoulder joint. Patients with scores <4 were classified into the Non-Shoulder Pain Group (NSPG), reflecting no pain or only minimal discomfort (McLaughlin et al., 2016; Temple et al., 2016).

#### 1.1.2 Clinical, epidemiological, and laboratory data

Consecutive patients with brucellosis were screened for eligibility. A total of 320 patients were initially assessed for inclusion, of whom 111 met the predefined inclusion criteria and were ultimately enrolled in the study, including 60 patients in the shoulder pain group (SPG) and 51 patients in the non–shoulder pain group (NSPG). Data collected included demographic characteristics, exposure history, clinical symptoms, and laboratory data. Laboratory tests included the Rose Bengal plate agglutination test and the standard serum agglutination test.

Blood cultures were performed in all patients; but were not used as primary diagnostic criteria due to low sensitivity. Molecular biological tests were performed in a subset of patients but were not required for inclusion due to limited availability.

This study received ethical approval from the institutional review board, and all participants signed informed consent prior to enrollment.

### 1.2 Ultrasonographic evaluation

#### 1.2.1 Equipment and protocol

Ultrasound examinations were conducted using a high-frequency color Doppler system (Philips Innosight, probe frequency 5–14 MHz). Scanning was performed with the patient seated and fully relaxed, following the *Chinese Guidelines for Musculoskeletal Ultrasound Examination* (2017) (Ultrasound Physician Branch of Chinese Medical Doctor Association, 2017). In the SPG, the affected side was examined; in the NSPG, the dominant side was selected.

The evaluation focused on the following anatomical structures: the long head of the biceps tendon (LHBT), rotator cuff, the subacromial-subdeltoid bursa (SASD), the glenohumeral capsule, and the proximal humeral cortex (PHC). Each site was scanned in longitudinal and transverse planes, and measurements were averaged. Dynamic scanning was used to assess tendon integrity.

#### 1.2.2 Imaging criteria

1. Bursal thickening was defined as SASD thickness ≥2.2 mm (data-derived cutoff). Thickening of the inferior glenohumeral capsule (measured at the axillary recess) was defined as a thickness >3.5 mm (Shrestha-Taylor et al., 2023; Su et al., 2024). Thickening, irregular margins, and heterogeneous echotexture were considered abnormal.
2. Rotator cuff hypervascularity was defined as the presence of ≥3 Doppler flow signals, or the presence of a focal or diffuse area of increased Doppler signal within any of the rotator cuff tendons (supraspinatus, infraspinatus, subscapularis, and teres minor) or their adjacent peritendinous tissues.
3. Effusion was defined as anechoic fluid ≥2 mm in the SASD bursa or LHBT sheath. LHBT sheath effusion was measured in the transverse plane at the bicipital groove, with additional assessment within 1 cm proximally and distally to identify the maximal thickness, ensuring standardized yet comprehensive evaluation (Park et al., 2015; Dammeyer et al., 2022).
4. PHC abnormality was defined as irregularity, discontinuity, or focal defects of the hyperechoic cortical line at the greater and lesser tubercles and anatomical neck, suggestive of cortical bone erosion (Cipolletta et al., 2021).

In total, six ultrasonographic variables were assessed, SASD bursal thickness, capsular thickness, rotator cuff hypervascularity, SASD effusion, LHBT sheath effusion, and PHC abnormality. All imaging features were recorded as binary variables (yes/no) for statistical analysis. The coracohumeral ligament was also evaluated during the preliminary assessment. However, as no significant difference was observed between groups in the pilot analysis, it was not included as a variable in the final statistical analysis.

### 1.3 Study design

Participants were divided into SPG and NSPG based on the presence or absence of shoulder pain. Ultrasound examinations were performed by two certified sonographers who had received standardized training and were blinded to pain grouping. All ultrasound images were independently reviewed by two experienced musculoskeletal sonographers using a standardized evaluation protocol based on the Chinese Guidelines for MSK US. In cases of disagreement, a senior MSK US expert with over 15 years of experience performed a final review, and consensus was reached to ensure consistency of sonographic evaluation.

### 1.4 Statistical analysis

All statistical analyses were performed using SPSS 27.0 and GraphPad Prism 9.5.1. The Shapiro–Wilk test was used to assess the normality of continuous variables. Normally distributed data were expressed as mean ± SD and compared using independent-sample t-tests; non-normally distributed data were reported as median (IQR) and analyzed with the Mann–Whitney U test. Categorical variables were compared using the χ^2^ test.

Ultrasound features showing significant intergroup differences (P < 0.05) were further tested for correlation with shoulder pain. Variables with significant associations were subjected to univariate logistic regression analysis. Significant variables were included in a Partial Least Squares Regression model to identify key predictors.

A nomogram was constructed to visualize the final predictive model. Model calibration was evaluated using the Hosmer–Lemeshow goodness-of-fit test. Discrimination was assessed by the area under the ROC curve (AUC), and clinical utility was compared using Decision Curve Analysis (DCA). A two-sided P < 0.05 was considered statistically significant.

## 2 Results

### 2.1 Descriptive statistics and baseline characteristics

A total of 111 brucellosis patients were included: 80 males (72.1%) and 31 females (27.9%), with a mean age of 43.5 ± 8.5 years. The majority (86.5%) had direct exposure to sheep, and 54.1% (n = 60) were herdsmen. Occupational exposure also included workers in slaughterhouses (14.4%), food handlers, veterinarians, and transport workers. Non-occupational exposure (18.9%) was mostly related to consumption of contaminated dairy or meat. Acute and chronic brucellosis were classified based on symptom duration, with acute disease defined as symptom duration <6 months and chronic disease defined as ≥6 months.

Clinical manifestations were consistent with brucellosis, with musculoskeletal symptoms being the most prevalent. No significant differences in baseline characteristics were observed between the SPG and NSPG groups (**Table 1**), indicating comparability between groups.

**Table 1:** Comparison of basic characteristics between brucellosis patients with and without shoulder pain.

Basic characteristics	n=111	NSPG (n=51)	SPG (n=60)	Z/χ^2^	P
Age [years, Median (IQR)]	46 (38, 51)	44(37, 51)	46(39, 50)	-1.18	0.236
Gender, n (%)					
- Female	31 (27.93)	14 (27.45)	17 (28.33)	0.01	0.918
- Male	80 (72.07)	37 (72.55)	43 (71.67)		
Occupational Exposure, n (%)					
- No	21 (18.92)	12 (23.53)	9 (15.00)	1.31	0.253
- Yes	90 (81.08)	39 (76.47)	51 (85.00)		
Acute/Chronic, n (%)					
- Chronic	47 (42.34)	18 (35.29)	29 (48.33)	1.92	0.166
- Acute	64 (57.66)	33 (64.71)	31 (51.67)		
Fever, n (%)					
- No	38 (34.23)	16 (31.37)	22 (36.67)	0.34	0.558
- Yes	73 (65.77)	35 (68.63)	38 (63.33)		
Fatigue, n (%)					
- No	44 (39.64)	24 (47.06)	20 (33.33)	2.17	0.141
- Yes	67 (60.36)	27 (52.94)	40 (66.67)		
Sweating, n (%)					
- No	33 (29.73)	14 (27.45)	19 (31.67)	0.23	0.628
- Yes	78 (70.27)	37 (72.55)	41 (68.33)		
Headache, n (%)					
- No	87 (78.38)	42 (82.35)	45 (75.00)	0.88	0.348
- Yes	24 (21.62)	9 (17.65)	15 (25.00)		
Weight Loss, n (%)					
- No	76 (68.47)	34 (66.67)	42 (70.00)	0.14	0.706
- Yes	35 (31.53)	17 (33.33)	18 (30.00)		
Appetite Loss, n (%)					
- No	84 (75.68)	38 (74.51)	46 (76.67)	0.07	0.792
- Yes	27 (24.32)	13 (25.49)	14 (23.33)		
Family History of Disease, n (%)					
- No	83 (74.77)	42 (82.35)	41 (68.33)	2.87	0.090
- Yes	28 (25.23)	9 (17.65)	19 (31.67)		

Z, Mann-Whitney test; χ^2^, Chi-square test.

### 2.2 Ultrasonographic findings between groups

**Figure 1:**
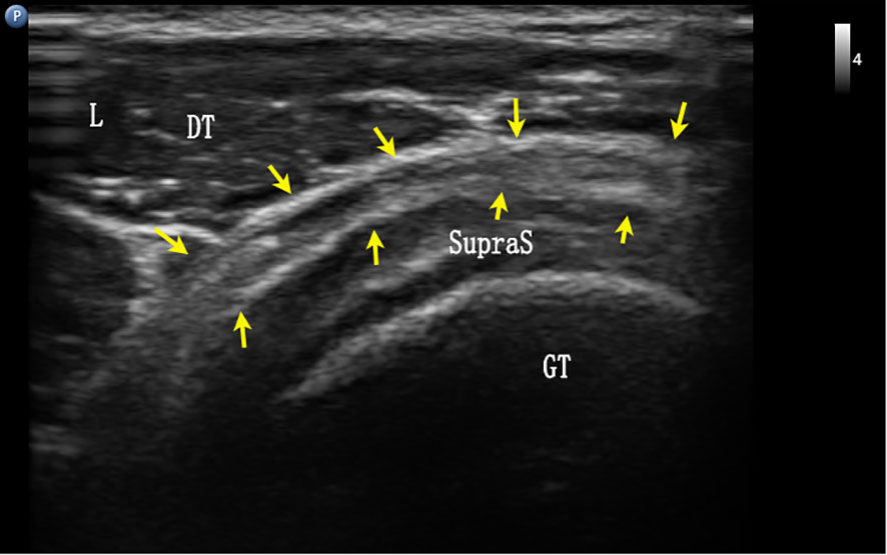
Thickening of the SASD bursa.

**Figure 2:**
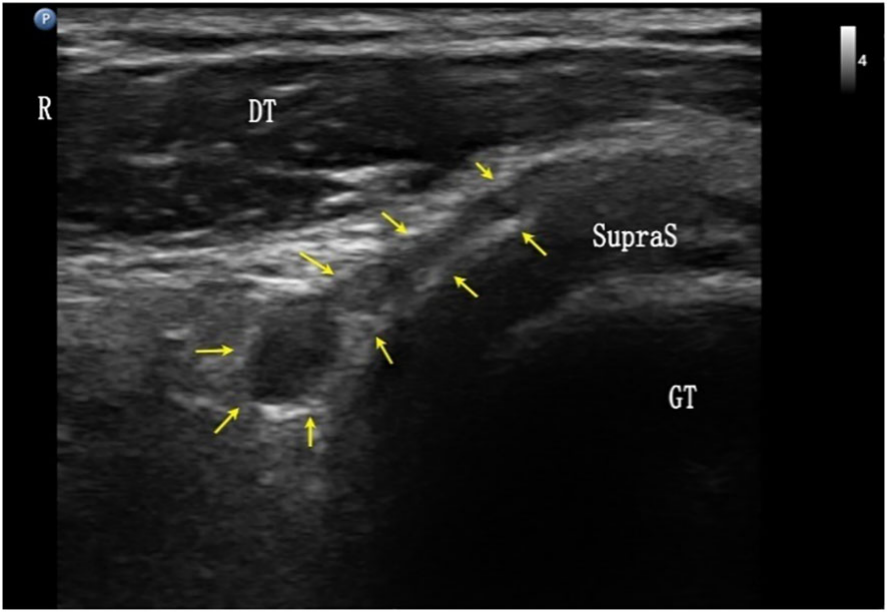
Effusion in the SASD bursa.

**Figure 3:**
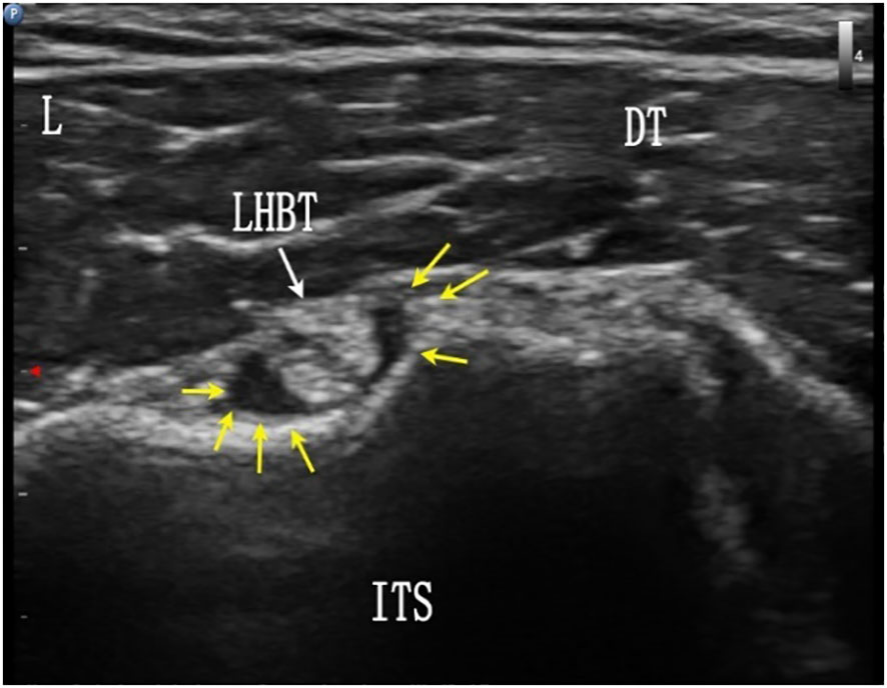
Effusion of the LHBT sheath.

**Figure 4:**
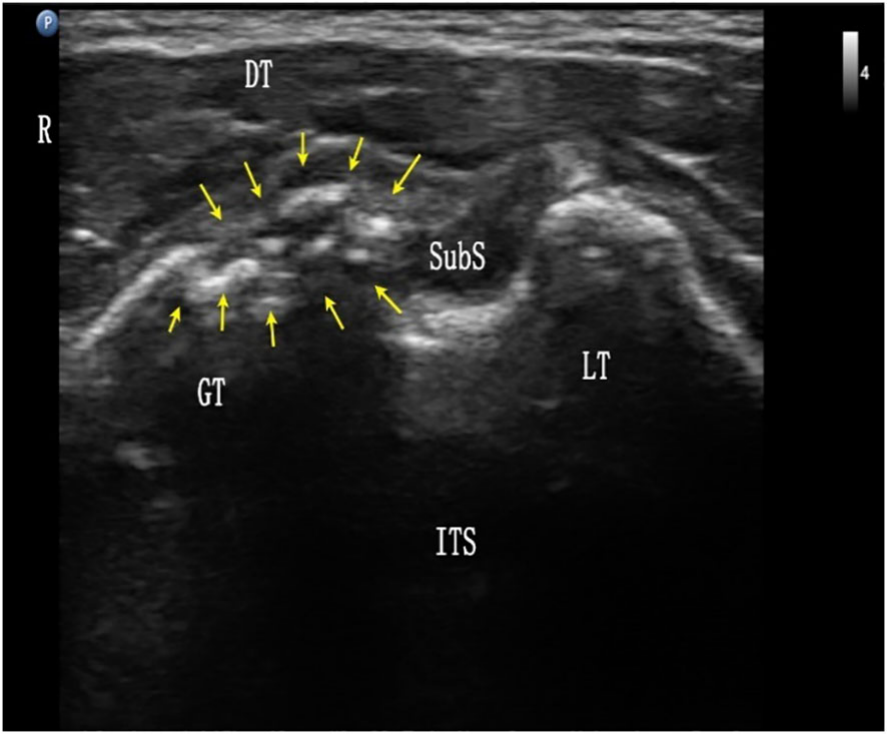
PHC abnormality.

**Figure 5:**
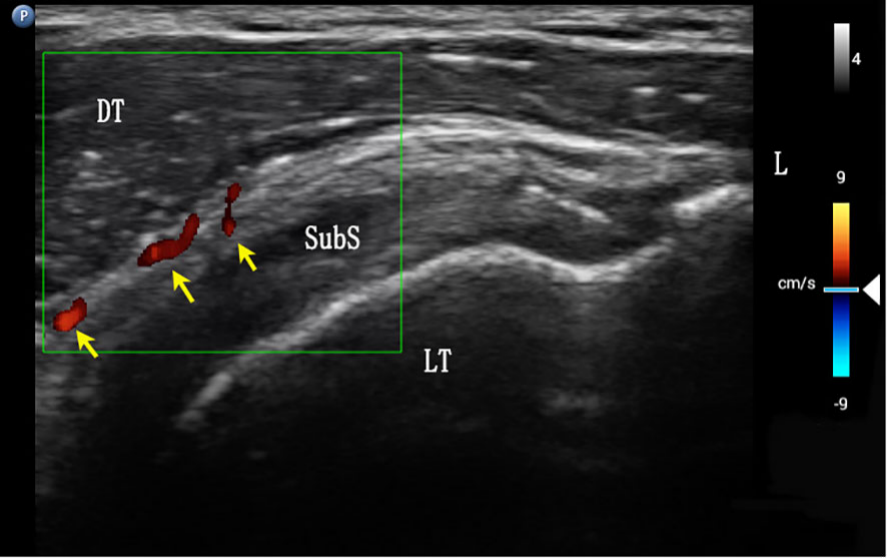
Increased synovial blood flow around the SubS tendon.

**Figure 6:**
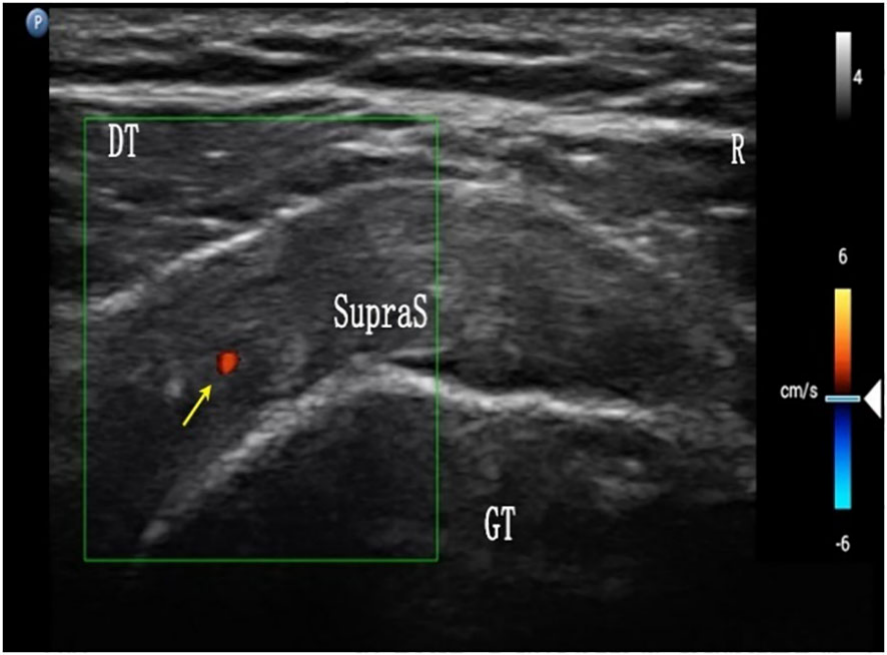
Increased intratendinous blood flow in the SupraS tendon.

Compared with the NSPG group, the SPG group showed significantly higher rates of SASD bursal effusion, LHBT tendon sheath effusion, rotator cuff hypervascularity, and PHC abnormalities. SASD bursal thickness was also significantly greater in the SPG group, whereas glenohumeral capsule thickness showed no significant difference (**Table 2**); representative images in **Figures 1**–**6**.

**Table 2:** Comparison of ultrasonographic features between shoulder pain and non-shoulder pain groups.

Ultrasonographic feature	Category	NSPG (n = 51)	SPG (n = 60)	Z/χ^2^	P
SASD bursal effusion, n (%)	No	48 (94.1%)	25 (41.7%)	31.396	P < 0.001
	Yes	3 (5.9%)	35 (58.3%)		
LHBT tendon sheath effusion, n (%)	No	43 (84.3%)	19 (31.7%)	30.989	P < 0.001
	Yes	8 (15.7%)	41 (68.3%)		
Rotator cuff hypervascularity, n (%)	No	45 (88.2%)	37 (61.7%)	10.083	P < 0.001
	Yes	6 (11.8%)	23 (38.3%)		
PHC abnormality, n (%)	No	43 (84.3%)	40 (66.7%)	4.551	0.033
	Yes	8 (15.7%)	20 (33.3%)		
SASD bursal thickness [mm, median (IQR)]		1.9 (1.8, 2.1)	2.5 (2.4, 2.7)	–8.454	P < 0.001
Glenohumeral capsule thickness [mm, median (IQR)]		2.4 (2.3, 2.6)	2.4 (2.2, 2.7)	–0.603	0.546

Z, Mann-Whitney test; χ^2^, Chi-square test.

### 2.3 Correlation between ultrasonographic features and shoulder pain

Correlation analysis was performed using Pearson’s correlation coefficient for continuous variables and the phi coefficient for categorical variables. SASD bursal thickness, as a quantitative variable, was analyzed using Pearson correlation, while other qualitative features were assessed using the phi coefficient.

Correlation analysis revealed a strong positive relationship between SASD bursal thickness and shoulder pain. Moderate correlations were observed for SASD bursal effusion and LHBT effusion, while rotator cuff hypervascularity and PHC abnormality showed significant but weak correlations. Detailed coefficients are presented in **Table 3**.

**Table 3:** Correlation between ultrasonographic features and the occurrence of shoulder pain.

Feature	Correlation coefficient (r/Phi)	P
SASD bursal thickness	0.750	P < 0.001
SASD bursal effusion	0.551	P < 0.001
LHBT tendon sheath effusion	0.528	P < 0.001
Rotator cuff hypervascularity	0.301	P < 0.001
PHC abnormality	0.202	0.033

### 2.4 Univariate logistic regression analysis

Univariate logistic regression showed that SASD bursal thickness (per 0.2 mm increment), SASD bursal effusion, LHBT effusion, rotator cuff hypervascularity, and PHC abnormality were all significantly associated with shoulder pain (P < 0.05), indicating a meaningful influence on pain occurrence. These variables were retained for multivariate modeling. Regression coefficients and odds ratios are presented in **Table 4**.

**Table 4:** Summary of univariate logistic regression analysis for ultrasonographic features associated with.

Feature	Coefficient	Wald χ^2^	P	OR	95% CI
SASD bursal thickness (per 0.2 mm increase)	12.11	5.10	P < 0.001	11.269	4.442-28.588
SASD bursal effusion (present)	3.109	22.866	P < 0.001	22.400	6.263-80.110
LHBT tendon sheath effusion (present)	2.451	26.664	P < 0.001	11.599	4.575-29.405
Rotator cuff hypervascularity (present)	1.539	9.137	P < 0.001	4.662	1.718-12.651
PHC abnormality (present)	0.989	4.378	0.036	2.687	1.065-6.785

### 2.5 Multivariate modeling with PLSR

To address multicollinearity, PLSR was performed using five ultrasound features to predict shoulder pain, selecting two principal components (**Table 5**). SASD bursal thickness was binarized (≥2.2 mm vs. <2.2 mm) based on ROC analysis.

**Table 5:** Principal component analysis for model performance evaluation.

Evaluation method	Intercept	Component 1	Component 2	Component 3	Component 4	Component 5
Cross-validation	1.01	0.6012	0.5854	0.5790	0.5771	0.5773
Adjusted cross-validation	1.01	0.6011	0.5852	0.5788	0.5769	0.5772

The PLSR model demonstrated good fit and predictive capacity (R^2^y = 0.693, Q^2^ = 0.661). SASD bursal thickness, SASD effusion, LHBT effusion, and PHC abnormality remained significant predictors, whereas rotator cuff hypervascularity did not contribute significantly to the model (**Table 6**).

**Table 6:** Regression analysis between ultrasonographic features and shoulder pain occurrence.

Ultrasonographic feature	Coefficient	Standardized coefficient	Standard error	T	P
SASD bursal thickness (≥2.2 mm)	0.557	0.547	0.126	5.781	P < 0.001
SASD bursal effusion (present)	0.185	0.176	0.150	2.392	0.018
LHBT tendon sheath effusion (present)	0.182	0.182	0.169	2.442	0.016
Rotator cuff hypervascularity (present)	0.084	0.074	0.157	1.086	0.280
PHC abnormality (present)	0.209	0.182	0.259	3.005	P < 0.001

Variables with VIP scores > 1 (SASD bursal thickness, SASD effusion, and LHBT effusion) were selected for the simplified model (**Table 7**).

**Table 7:** Summary of variable importance in projection (VIP) scores based on two principal components.

Ultrasonographic feature	VIP score
SASD bursal thickness (≥2.2 mm)	1.534
SASD bursal effusion (present)	1.055
LHBT tendon sheath effusion (present)	1.005
Rotator cuff hypervascularity (present)	0.590
PHC abnormality (present)	0.418

### 2.6 ROC curve analysis of predictive models

A three-variable model was constructed using variables with VIP scores > 1 (SASD bursal thickness, SASD bursal effusion, and LHBT effusion). Two additional variables (rotator cuff hypervascularity and PHC abnormality), identified based on statistically significant intergroup differences, were added to build a five-variable model. A single-variable model based on SASD bursal thickness was also evaluated.

**Figure 7:**
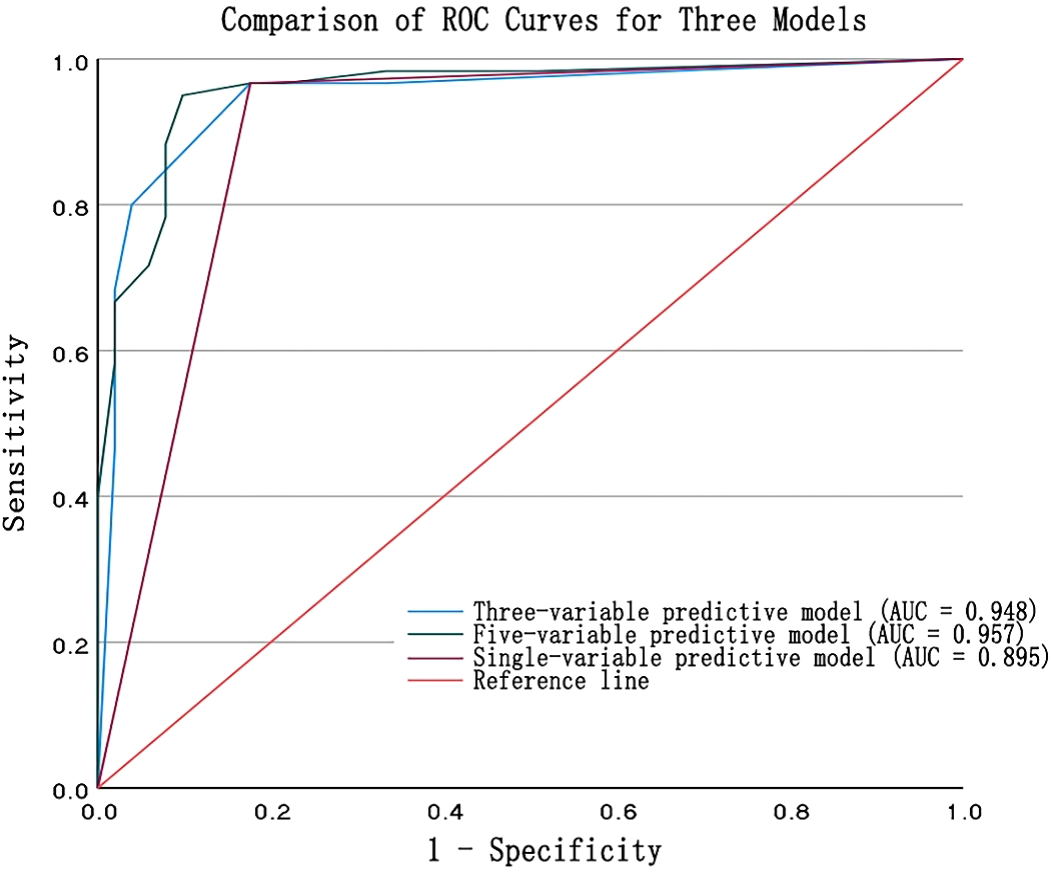
Comparison of ROC curves for the three predictive models.

ROC analysis demonstrated that both multi-variable models achieved high diagnostic performance, with the five-variable model showing higher specificity and the three-variable model higher sensitivity (**Table 8**, **Figure 7**). No significant difference in AUC was observed between the two models (P > 0.05), whereas both outperformed the single-variable model (P < 0.05) (**Table 9**).

**Table 8:** Summary of ROC analysis for predictive models.

Model type	AUC	Standard error	P	95% CI	Optimal cutoff	Sensitivity	Specificity
Three-variable model	0.948	0.023	P < 0.001	0.903 – 0.992	0.79	0.967	0.824
Five-variable model	0.957	0.019	P < 0.001	0.919 – 0.994	0.852	0.950	0.902
Single-variable model	0.895	0.035	P < 0.001	0.827 – 0.963	0.79	0.967	0.824

**Table 9:** Pairwise comparison of AUC values using DeLong test.

Model 1	Model 2	AUC difference	Standard error	95% CI	Z	P
Three-variable model	Five-variable model	0.0093	0.0112	–0.013-0.031	0.828	0.408
Three-variable model	Single-variable model	0.0525	0.0216	0.010-0.095	2.429	0.015
Five-variable model	Single-variable model	0.0618	0.0216	0.019-0.104	2.858	P < 0.001

From a clinical perspective, the five-variable model may be more suitable when a higher specificity is required to confirm shoulder involvement and reduce false-positive diagnoses, while the three-variable model, with its higher sensitivity and simpler structure, may be preferable for initial screening and early detection in routine clinical settings.

### 2.7 Nomogram construction and visualization

**Figure 8:**
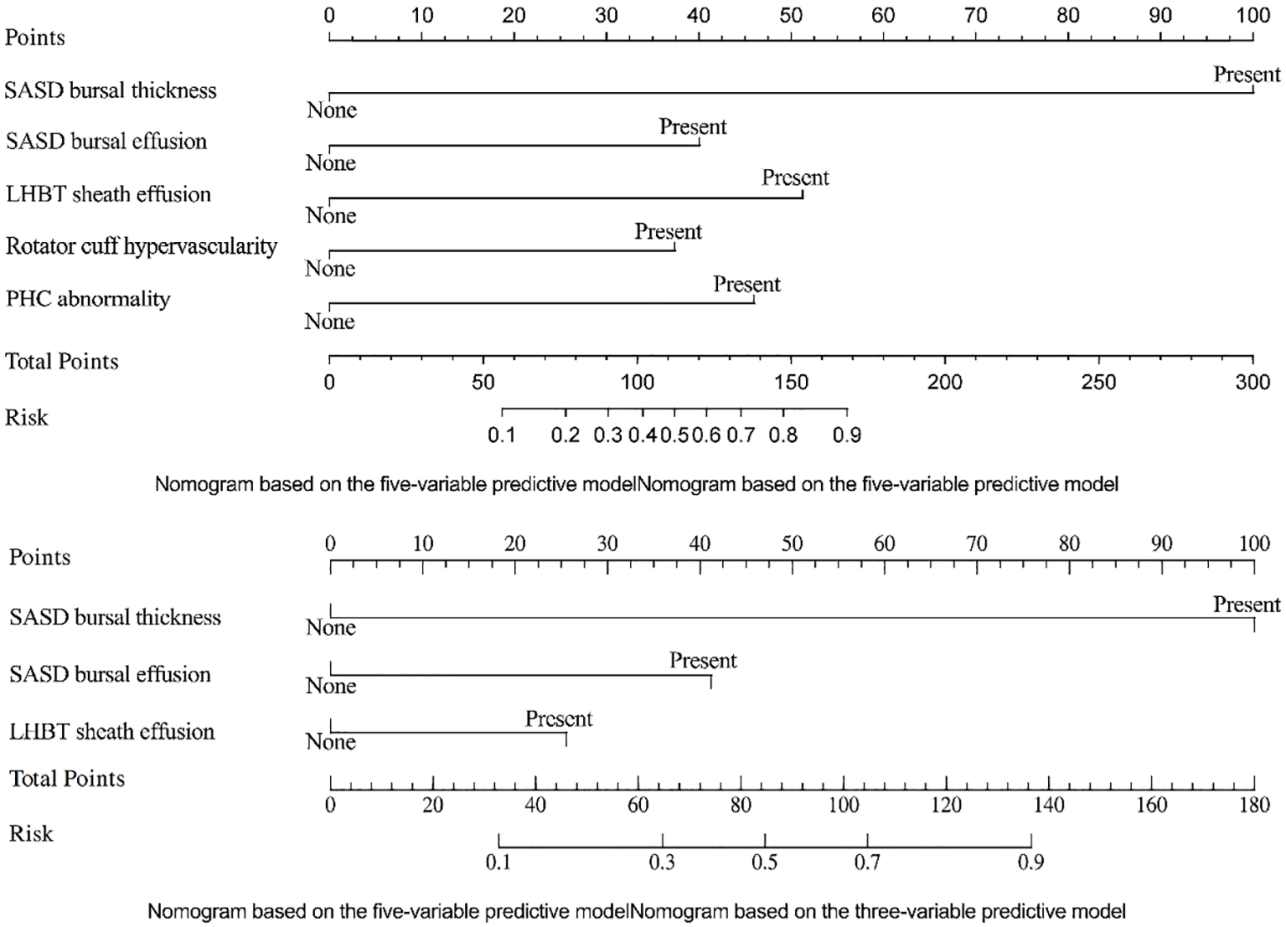
Nomograms constructed based on the five-variable (upper panel) and three-variable (lower panel) predictive models for brucellosis-related shoulder pain.

In this study, three ultrasound-based predictive models were developed to assess the likelihood of shoulder involvement in brucellosis. Based on DeLong’s test, the three-variable and five-variable models were retained for further analysis and visualization. Both were illustrated using PLS regression–based nomograms generated via the “rms” package in R (**Figure 8**).

In both models, SASD bursal thickness contributed the greatest weight, followed by LHBT effusion and SASD effusion, while rotator cuff hypervascularity and PHC abnormality showed relatively lower contributions in the five-variable model.

### 2.8 Internal validation

**Figure 9:**
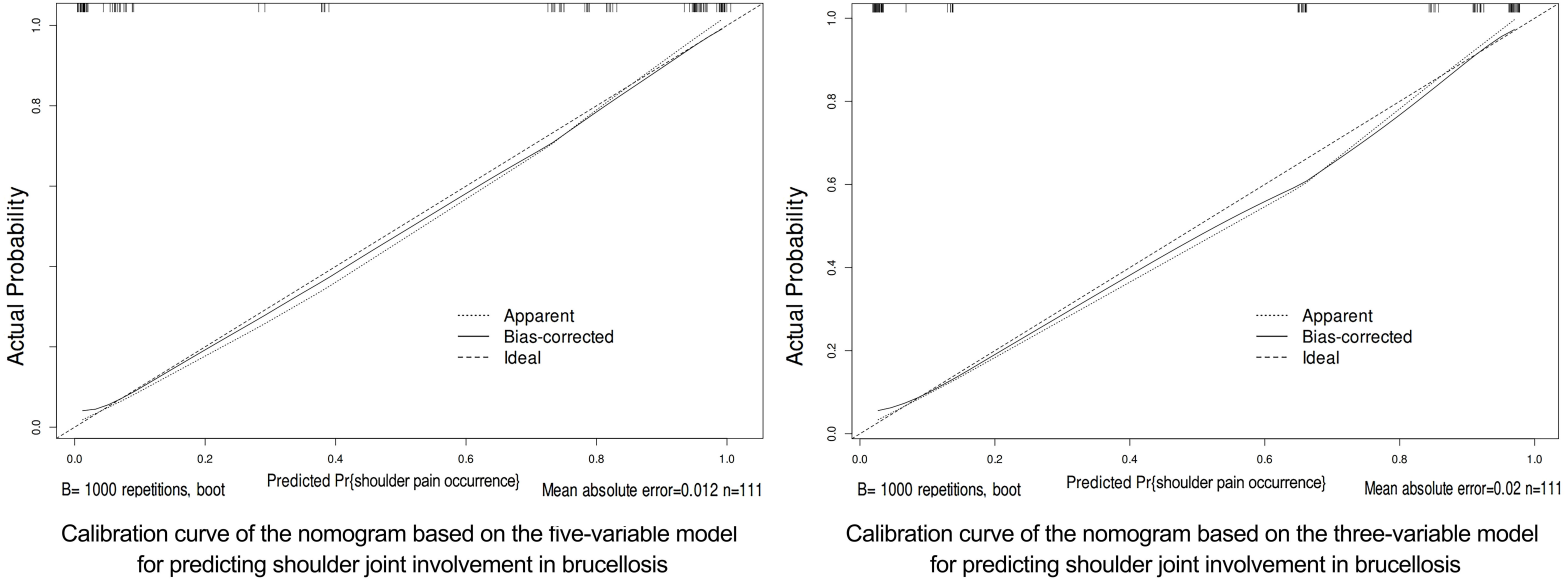
Calibration curves of the nomograms for predicting shoulder joint involvement in brucellosis.

Bootstrap validation with 1,000 resamples showed high consistency between predicted and actual outcomes. The three-variable model had a mean absolute error of 0.02 and passed the Hosmer–Lemeshow test (χ^2^ = 1.34, P = 0.5115). The five-variable model had a lower error (0.012) and also passed calibration (χ^2^ = 4.98, P = 0.5459), indicating better precision and robustness (**Figure 9**).

### 2.9 Clinical utility via decision curve analysis (DCA)

**Figure 10:**
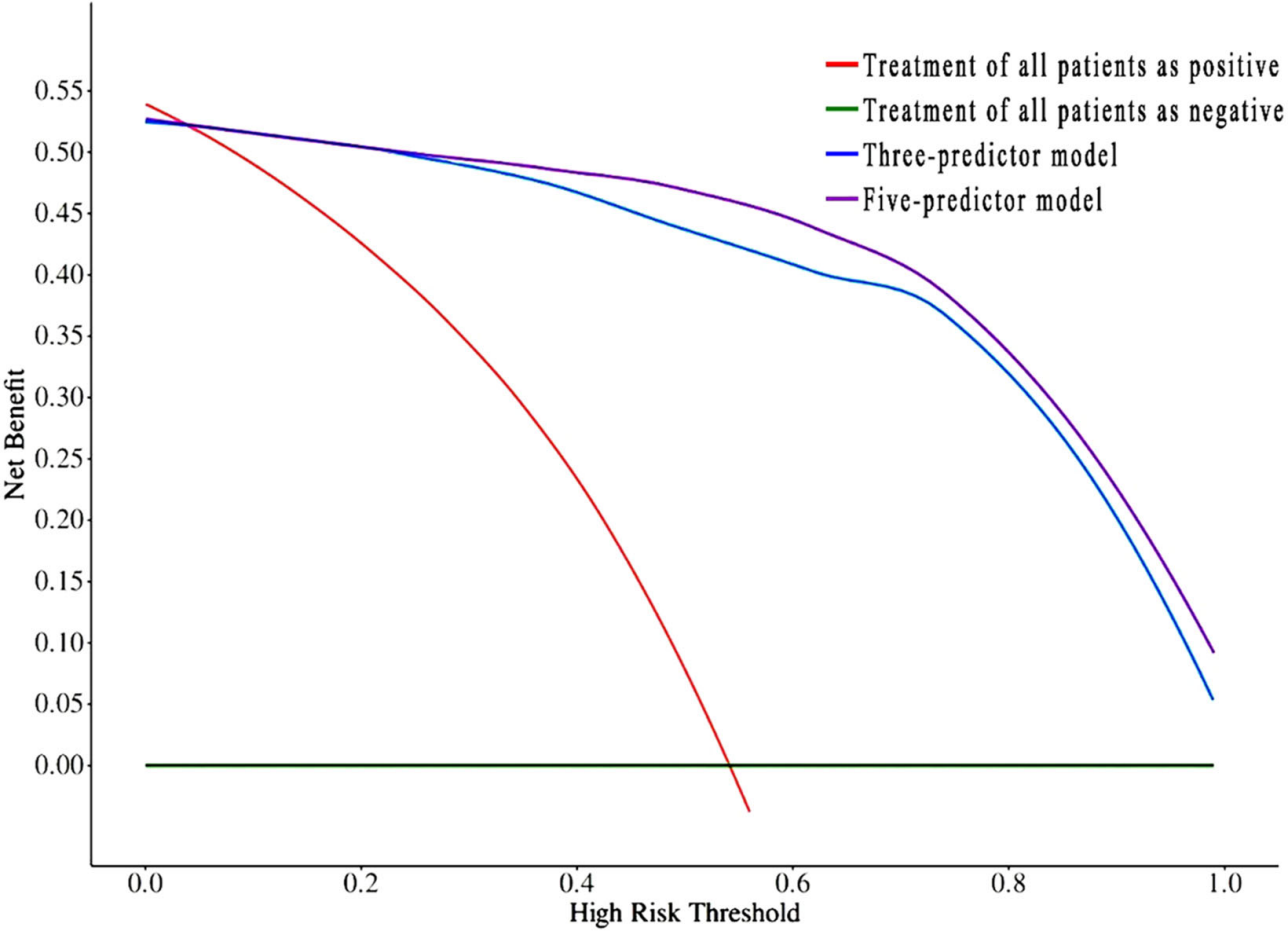
Decision curve analysis comparing the three-variable and five-variable predictive models.

DCA showed that both models provided net clinical benefit over the “treat all” and “treat none” strategies across a range of threshold probabilities (**Figure 10**). The five-variable model consistently demonstrated higher net benefit over a broader threshold range, whereas the three-variable model maintained favorable performance with a simpler structure.

In clinical application, predicted probabilities derived from the nomogram (**Figure 8**) can be interpreted in relation to a clinician’s chosen threshold probability for intervention. When the predicted probability exceeds this threshold, clinical intervention may be considered.

Within this framework, the three-variable model is particularly suitable for initial assessment and triage, especially in settings where lower threshold probabilities are preferred to minimize under-diagnosis. In contrast, the five-variable model provides superior net benefit at higher threshold probabilities, supporting its use in guiding confirmatory evaluation or decisions regarding more intensive or invasive therapeutic strategies.

## 3 Discussion

This study systematically explored the association between ultrasonographic features and shoulder pain in patients with brucellosis-related shoulder periarthritis and developed diagnostic prediction models to assess their clinical utility. The findings underscore the diagnostic significance of MSK US and provide a foundation for early detection and individualized management strategies.

### 3.1 Baseline characteristics and epidemiological insight

The cohort included 111 patients (80 males, 31 females; age range: 25–55 years; mean age: 43.5 years), with a male-to-female ratio of 2.6:1, consistent with the known epidemiological patterns of brucellosis (Yu et al., 2023). This age group was selected to minimize confounding from age-related degenerative shoulder conditions, allowing for a more accurate assessment of brucellosis-related involvement. Sheep were the most common exposure source (86.5%), and accordingly, most patients had a history of occupational exposure. Notably, 18.9% of patients reported no occupational contact, suggesting that non-occupational transmission routes—such as consumption of uninspected meat or dairy products—should also warrant attention in public health efforts. All patients were diagnosed based on the Chinese Diagnostic Criteria for Brucellosis (WS 269-2019), which rely on compatible clinical manifestations and positive serological tests. Although blood cultures and molecular diagnostic methods were available in a subset of patients, their limited sensitivity and accessibility prevented their use as universal diagnostic criteria. Therefore, they were not used as inclusion requirements.

### 3.2 Clinical manifestations and study design consideration

Common symptoms included fever (66%), fatigue (60%), hyperhidrosis (70%), headache (22%), and musculoskeletal pain (90%), consistent with Brucella’s osteoarticular tropism. Although shoulder involvement is less common than sacroiliac or knee arthritis, this study focused on patients with shoulder symptoms to enable detailed ultrasonographic analysis. Therefore, the proportion of shoulder involvement observed should not be interpreted as reflecting its true population prevalence (Adetunji et al., 2019).

This may be partly attributable to the tendency for diffuse and migratory musculoskeletal pain during the acute phase of brucellosis to mask localized shoulder involvement, and partly to the lack of routine screening of the shoulder using dedicated imaging modalities such as MSK US or magnetic resonance imaging in this patient population. Moreover, specific local treatment strategies targeting shoulder involvement in brucellosis remain insufficiently defined, which may predispose a subset of patients to persistent inflammation or progression to more severe local pathology, including suppurative periarticular involvement (Trabelsi et al., 2022).

Importantly, while no pathognomonic ultrasonographic features specific to brucellosis-related shoulder disease were identified, the present study suggests that inflammatory involvement in brucellosis tends to extend across multiple anatomical components within the shoulder complex rather than being confined to a single structure. This pattern of multi-compartmental involvement within the shoulder underscores the value of comprehensive MSK US assessment. Accordingly, the study design prioritized analytical depth and statistical power to explore these imaging patterns, rather than to estimate the population-level prevalence of shoulder involvement in brucellosis.

### 3.3 Key ultrasonographic features correlated with shoulder pain

Ultrasound revealed characteristic pathological features—primarily SASD bursa thickening, SASD bursal effusion, LHBT tendon sheath effusion, increased rotator cuff hypervascularity, and PHC abnormality. Among all features, SASD bursal thickness (≥2.2 mm) demonstrated the strongest correlation with pain (r = 0.750), with both statistical significance and a high VIP score (1.534) supporting its predictive value. This may reflect synovial edema and inflammatory infiltration caused by Brucella-induced immune responses, resulting in mechanical compression and nociceptive stimulation (Grey et al., 2023).

SASD bursal effusion and LHBT tendon sheath effusion also showed moderate positive correlations (Phi = 0.551 and 0.528, respectively), likely reflecting increased synovial permeability secondary to infection (Weaver et al., n.d.). Aspiration of these effusions may yield Brucella spp., although the positive rate remains low. Clinically, persistent or large-volume effusions should raise concern for progression to suppurative arthritis, underscoring the importance of timely evaluation and potential aspiration (Chernchujit et al., 2022).

Rotator cuff hypervascularity and PHC abnormality showed weaker associations but remain relevant, especially in atypical or advanced cases. The former suggests inflammatory hyperemia (Xiong et al., 2025), while the latter may represent early osteitis or cortical erosion (Giambartolomei et al., 2017).

Brucellosis-related shoulder periarthritis may be misdiagnosed as nonspecific bursitis, particularly in the absence of clear epidemiological history (Wang et al., 2021). Therefore, in endemic areas, clinicians and sonographers should remain vigilant, and ultrasonography should be emphasized as a valuable tool for early diagnosis and treatment planning in musculoskeletal brucellosis.

### 3.4 Differential diagnosis considerations

Brucellosis-related shoulder periarthritis may be clinically indistinguishable from adhesive capsulitis (Le et al., 2017; Wang et al., 2021). However, systemic symptoms (fever, fatigue) and laboratory abnormalities (elevated ESR/CRP) (Balın et al., 2018) favor infectious etiology. Ultrasonographically, brucellosis is more likely to show bursal thickening/effusion and tendon sheath involvement, while adhesive capsulitis primarily affects the glenohumeral capsule and coracohumeral ligament. Comprehensive imaging and laboratory assessment are thus critical for differential diagnosis, especially in endemic areas. In the present study, thickness of the glenohumeral capsule was evaluated and showed no significant difference between groups. The coracohumeral ligament was also assessed during the preliminary analysis, but was not included in the final model due to a lack of significant intergroup difference. These findings suggest that capsular and ligamentous involvement may be less prominent in brucellosis-related shoulder involvement compared with adhesive capsulitis.

Other common causes of shoulder pain, including degenerative or mechanical disorders such as isolated SASD bursitis or rotator cuff tendinopathy, typically present with localized abnormalities confined to a single anatomical compartment, most often bursal thickening or focal tendon degeneration, with limited extra-structural involvement. Traumatic shoulder conditions likewise tend to demonstrate focal structural damage corresponding to the site of injury, such as partial- or full-thickness tendon tears or localized cortical irregularities, rather than diffuse inflammatory changes.

In contrast, brucellosis is a systemic infectious disease with a well-recognized propensity for osteoarticular dissemination. When the shoulder is involved, inflammatory changes frequently extend across multiple tissue compartments, including bursae, tendon sheaths, peri-tendinous soft tissues, and even the cortical bone surface. Accordingly, the diagnostic value of MSK US in this setting lies not in identifying a single disease-specific imaging feature, but in recognizing the coexistence of inflammatory abnormalities across several anatomical structures. When such a multi-compartmental pattern is observed, particularly in patients with compatible systemic manifestations or epidemiological risk factors, brucellosis should be considered in the differential diagnosis, even when individual ultrasonographic findings overlap with those of more common shoulder disorders.

### 3.5 Methodological considerations and comparison with existing models

In this study, PLS regression was employed to address multicollinearity among ultrasonographic features—a common challenge when evaluating closely related inflammatory signs such as bursal thickness and effusion. This methodological choice differs from the conventional multivariable logistic regression used in recent MSK US prediction models, including those for rotator cuff retear (Hu et al., 2025) and rheumatoid arthritis bone erosion (Yan et al., 2025). By extracting latent components that maximize covariance between predictors and the outcome, PLS regression ensures model stability even when imaging features are highly intercorrelated, offering a complementary rather than competitive approach. Beyond musculoskeletal conditions, predictive modeling for brucellosis has largely relied on laboratory parameters to assess systemic chronicity. A recent machine learning study demonstrated the clinical utility of such an approach using DCA (Wang et al., 2025). While that investigation focused exclusively on serological and biochemical markers, our work complements it by demonstrating that ultrasound imaging features alone can provide clinically meaningful predictions for focal shoulder involvement. Taken together, imaging and laboratory data appear to capture different yet complementary dimensions of the same disease process. Future research integrating both modalities—combining MSK US with serological markers—may further enhance predictive performance, warranting investigation in larger, prospective cohorts with external validation.

### 3.6 Clinical application and practical considerations

The clinical utility of our models was confirmed by DCA across a range of threshold probabilities. As detailed previously, the three-variable model offers higher sensitivity and simplicity, making it well suited for rapid screening in primary care or resource-limited settings. Conversely, the five-variable model, with better specificity, is more appropriate for diagnostic confirmation and severity assessment in tertiary centers. This flexible, risk-stratified approach allows clinicians to choose between models based on the specific clinical scenario and their threshold for intervention. By translating ultrasound features into quantifiable predictive tools, our findings support evidence-based decision-making for early intervention, potentially reducing diagnostic delays and preventing irreversible joint damage in patients with brucellosis.

### 3.7 Trade-off between model complexity and clinical feasibility

An additional consideration is the time and complexity required for model implementation. Compared with the three-variable model, the five-variable model requires two additional assessments: rotator cuff hypervascularity and PHC abnormality.

Rotator cuff hypervascularity requires Doppler evaluation of four distinct anatomical sites (supraspinatus, infraspinatus, subscapularis, and teres minor tendons) and their surrounding peritendinous tissues, often requiring multiple transducer repositionings and adjustments of Doppler settings. PHC abnormality requires dynamic scanning and careful analysis of the cortical contour across the greater tubercle, lesser tubercle, and anatomical neck—a broader, deeper, and more technically demanding examination.

In contrast, the three-variable model focuses on SASD bursal thickness, SASD bursal effusion, and LHBT tendon sheath effusion. The two SASD parameters are assessed simultaneously at a single location, and LHBT evaluation is confined to the bicipital groove and its immediate proximal/distal segments (within 1 cm). These features are more localized, easier to identify, and require less scanning time and operator expertise.

Although the exact time difference varies by operator experience, the additional steps required for the five-variable model—Doppler imaging across multiple sites, extended scanning range, and detailed cortical analysis—constitute a substantial portion of the total examination time. Therefore, while the five-variable model offers higher specificity and net clinical benefit, its time cost may not be justified in all settings. In high-throughput or screening scenarios (e.g., primary care or field settings), the three-variable model is more practical for rapid triage. In contrast, the five-variable model is better suited for tertiary care settings where comprehensive evaluation is needed to confirm diagnosis or guide intensive therapy.

### 3.8 Application of MSK US in the management of brucellosis-related shoulder periarthritis

The ultrasonographic features identified in this study offer value beyond diagnosis, providing practical guidance for the management of Brucella-related shoulder periarthritis. SASD bursal thickening and effusion, as well as rotator cuff hypervascularity, often indicate active shoulder inflammation, prompting closer monitoring of treatment response and consideration of extending or adjusting antimicrobial therapy in cases of persistent inflammation. For patients with ongoing symptoms or at risk of suppurative progression, ultrasound can guide individualized local interventions, such as intra-bursal antibiotic injection for markedly thickened bursae or ultrasound-guided joint capsule distension for restricted shoulder mobility. Detection of SASD or LHBT tendon sheath effusion also provides an objective indication for ultrasound-guided aspiration, which allows specimen collection to exclude suppurative evolution and relieves pressure-related pain. PHC abnormality may suggest prolonged inflammation with early structural involvement, warranting follow-up imaging with MRI or CT and timely clinical reassessment. Collectively, these ultrasonographic indicators help identify patients who may benefit from aspiration, extended antimicrobial therapy, or targeted local interventions, supporting a more precise and individualized disease management strategy.

### 3.9 Study limitations and future directions

This single-center, cross-sectional study has several limitations, including limited sample size, lack of external validation, and potential observer variability in ultrasound interpretation. Although the lack of microbiological confirmation is a recognized limitation in musculoskeletal brucellosis research, the combined clinical–imaging criteria used in this study are well supported by existing literature. Additionally, the study focused on shoulder pathology without comparing other differential causes of shoulder pain (e.g., rotator cuff tears, septic arthritis, or tuberculosis-related arthritis), and the cross-sectional design limits assessment of the prognostic value of these ultrasound features.

Future research should therefore include larger, multi-center cohorts for prospective external validation, along with inter-observer reliability analysis and cost-effectiveness assessments. Longitudinal studies are needed to link early sonographic findings with treatment response and long-term outcomes. Comparative studies with other joint pathologies will also help broaden the utility of these models in differential diagnosis. Integration of artificial intelligence in ultrasound image interpretation may further enhance objectivity and scalability.

## 4 Conclusion

This study demonstrates that MSK US is a valuable tool in detecting brucellosis-related shoulder periarthritis. SASD bursa thickening, effusion, and LHBT tendon sheath effusion emerged as core imaging features associated with pain, which can aid in guiding local interventions and formulating individualized clinical management strategies. Based on these features, we developed and validated predictive models with strong clinical applicability. The three-variable model is well suited for preliminary screening, while the five-variable model offers higher specificity for comprehensive assessment.

These models enhance diagnostic precision, promote early intervention, and offer a practical strategy for integrating ultrasound into brucellosis management, especially in endemic and low-resource regions. Future multicenter validation will further establish their utility in diverse clinical settings.

## Data Availability

The original contributions presented in the study are included in the article/supplementary material. Further inquiries can be directed to the corresponding author.
